# Integrated Health and Social Care in the United States: A Decade of Policy Progress

**DOI:** 10.5334/ijic.5687

**Published:** 2021-10-29

**Authors:** Sahil Sandhu, Anu Sharma, Rushina Cholera, Janet Prvu Bettger

**Affiliations:** 1 Duke-Margolis Center for Health Policy, Duke University, NC, US; 2 Population Health Sciences Institute, Newcastle University, UK; 3 National Clinicians Scholars Program, Department of Pediatrics, School of Medicine, Duke University, NC, US; 4 Department of Orthopaedic Surgery, School of Medicine, Duke University, NC, US; 5 Duke Global Health Institute, Duke University, NC, US

**Keywords:** integrated care, health care reform, health policy, Medicaid, social determinants of health

## Abstract

**Introduction:**

Over the last decade in the United States (US), the burden of chronic disease, health care costs, and fragmented care delivery have increased at alarming rates. To address these challenges, policymakers have prioritized new payment and delivery models to incentivize better integrated health and social services.

**Policy practice:**

This paper outlines three major national and state policy initiatives to improve integrated health and social care over the last ten years in the US, with a focus on the Medicaid public insurance program for Americans with low incomes. Activities supported by these initiatives include screening patients for social risks in primary care clinics; building new cross-sector collaborations; financing social care with healthcare dollars; and sharing data across health, social and community services. Stakeholders from the private sector, including health systems and insurers, have partnered to advance and scale these initiatives. This paper describes the implementation and effectiveness of such efforts, and lessons learned from translating policy to practice.

**Discussion and Conclusion:**

National policies have catalyzed initiatives to test new integrated health and social care models, with the ultimate goal of improving population health and decreasing costs. Preliminary findings demonstrated the need for validated measures of social risk, engagement across levels of organizational leadership and frontline staff, and greater flexibility from national policymakers in order to align incentives across sectors.

## Introduction

Health in the United States (US) is remarkably poor given the personal and societal costs for healthcare. Despite spending nearly double of its gross domestic product on health care compared to the average Organization for Economic Co-operation and Development (OECD) country, the US has the highest chronic disease burden, highest rate of avoidable deaths, and lowest life expectancy among high-income countries [1]. This misalignment has sparked a range of policy reforms over the last ten years, including innovative efforts to reduce fragmentation, improve coordination across systems and sectors, and address the social determinants of health (SDOH).

The purpose of this paper is to describe national and state policy initiatives over the last ten years in the US aimed to integrate health and social care for Americans with health insurance provided by the public Medicaid insurance program. We also contextualize the US experience in the broader international landscape using an integrated care framework. Policy reform in the US can inform and be informed by other countries in an effort to globally advance integrated health and social care.

## Healthcare System Context

The US is a country of fifty states, a federal district, five major territories, multiple minor islands, and nearly 330 million people. The healthcare system is a mixture of public and private providers and healthcare insurers. In 2019, about 56% of Americans were covered through private insurance (mostly employer-sponsored); 35% were covered through public insurance (e.g., “Medicare” predominantly for older adults; “Medicaid” for Americans with low incomes; and other public programs for active military, veterans, American Indians, and Alaskan Natives), and 9% were uninsured [2].

Traditionally, health services in the US have been paid in a fee-for-service model. In this model, providers, office visits, tests, procedures, and treatments are each paid for separately. This structure incentivizes quantity of services rather than quality of care. In 2010, President Obama signed into law the Affordable Care Act, one of the most comprehensive healthcare reform policies in American history. The law aimed to achieve universal health care coverage, while controlling costs and improving quality of care through new payment structures [3]. The Affordable Care Act supported new value-based payment models in which payment for health care services is tied to quality and cost rather than volume [4]. Examples of value-based payment models include giving health systems global budgets to care for patient populations rather than linking payments to individual services for each patient, and making payments conditional on meeting specific quality measures [5]. Providers and insurers have financial flexibility in value-based payment models to develop creative solutions for improving access, coordination, and integration across settings and sectors.

Innovative approaches to integrate health and social services expanded rapidly with the shift towards value-based payment models and the growing evidence that adverse social determinants of health (SDOH) are major drivers of poor health. Research showing that health care alone only shapes 10–20% of an individual’s health status led to greater attention on upstream SDOH including economic stability, housing stability, neighborhood environment, educational attainment, and food security [6,7,8]. A meta-analysis concluded that the number of deaths in 2000 in the US attributable to low education, racial segregation, and low social support, were comparable to those attributed to myocardial infarctions, cerebrovascular disease, and lung cancer respectively [9]. In addition to impacts on mortality, research has demonstrated clear links between adverse SDOH and a multitude of outcomes, including health expenditures, healthcare utilization, physical health, mental health, chronic disease management, and health-related behaviors (e.g., exercise, diet, sleep, smoking, drug use) [7,10].

In response, the health sector expanded implementation and evaluation of a variety of integration efforts to identify and address individuals’ social risks and unmet social needs. Social risks refer to the adverse social conditions associated with an individual’s poor health, such as food insecurity and housing instability [10,11]. Social needs refer to the specific social risks that a patient perceives as most pressing to them. Both these terms are more precise than the broader term of social determinants of health which refer to a community’s underlying social and economic conditions that affect everyone and can both be positive or negative. Health system approaches to address social risks and needs have spanned from individual-level activities, such as screening patients for social risks and referring them to external social services, to community-level approaches, such as cross-sector technology referral platforms and health clinic-food bank partnerships [12]. In 2019, the National Academies of Sciences, Engineering, and Medicine released a consensus report proposing a framework for activities to integrate social care into healthcare delivery and outlining recommendations to guide future practice and policy efforts [13,14].

US government agencies, and the Medicaid program as part of the Centers for Medicare and Medicaid Services (CMS) in particular, have played a critical role in advancing integrated health and social care through policy and payment reform. The Medicaid insurance program is a public insurance program for people with low incomes, covering about 20% of Americans (70+ million) [15]. Specifically, Medicaid covers 49% of births, 38% of all children, 45% of non-elderly adults with disabilities, and 62% of nursing home residents [15]. Created in 1965, Medicaid is a jointly financed federal and state partnership, in which the federal government sets core requirements for eligibility and benefits, and individual states administer the program. The Affordable Care Act further expanded the Medicaid program to cover over 12 million additional Americans [16]. Given Americans covered by Medicaid face greater socioeconomic challenges compared to Americans with private insurance, and are traditionally at highest risk for unmet social needs and societal inequities, the Medicaid program has been uniquely poised and motivated to integrate social care into health care delivery.

## Recent Integrated Care Reforms in the United States Medicaid Program

**Figure 1:**
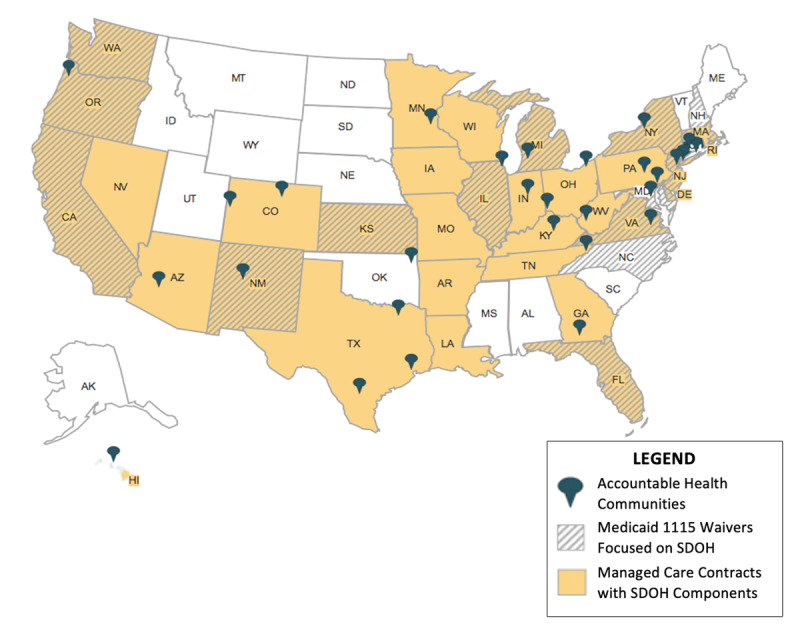
Map of Integrated Care Reforms in the US Medicaid Program.

Three major policy initiatives in the last decade aimed to advance integrated care and improve health for populations with Medicaid health insurance coverage. First, we describe the Accountable Health Communities demonstration program to integrate health, social and community-based organizations funded by the federal government for Medicaid recipients in selected regions. Then, we describe two policy tools leveraged by state Medicaid agencies to integrate health and social care: 1115 waivers, which give state Medicaid agencies flexibility to test novel approaches to service delivery and payment and managed care contracts, under which state Medicaid services are delivered by risk-based private insurance companies. ***Table 1*** provides an overview of each of the three integrated care reforms. ***Figure 1*** shows the geographic distribution of these integrated care programs and efforts.

**Table 1:** Overview of Integrated Health and Social Care Reforms in the US Medicaid Program.


REFORM	DESCRIPTION

Accountable Health Communities	Federally-funded model to systematically test social risk screening, referrals, and community navigation services to address social needs of Medicaid and Medicare beneficiaries.

Section 1115 Waivers Programs	Waiver that allows states to test new approaches in Medicaid that differ from federal requirements. In the last decade, states have applied to use waivers to implement new SDOH-related programs.

Managed Care Contracts	Contracts between state Medicaid agencies and private managed care organizations (MCOs) that deliver Medicaid services to beneficiaries. In the last decade, states have used contracts to require or incentivize MCOs to implement SDOH interventions.


### Accountable Health Communities

#### Description

The Affordable Care Act funded the new Center for Medicare and Medicaid Innovation (CMMI) to design and test innovations in payment and care delivery. In 2016, CMMI announced a five year, $157 million program to scale and evaluate the “Accountable Health Communities” model, which aims to address health-related social needs through improved coordinated efforts between health care organizations and community services [17]. CMMI funded 29 organizations in 21 states across the country to implement this model and bring together diverse stakeholders (***Figure 1***). Funded organizations varied in type, including county governments, hospitals, universities, and public health departments. CMMI provides organizations with funding and technical assistance, in addition to outlining program requirements.

The foundation of the model for all sites rested on universal and standardized screening of all Medicare and Medicaid beneficiaries for five social needs domains: housing, food insecurity, utility, interpersonal violence, and transportation. CMMI convened a Technical Expert Panel to create a standardized 10-question social needs screening tool to be used in the model [18].

Individual sites applied to participate in one of three tracks: “awareness,” “assistance” or “alignment.” Each track had distinct program requirements and funding amounts. Healthcare organizations in the awareness track were required to screen patients for social needs and refer patients to community services. Those in the assistance track provided intensive community navigation (e.g., follow-up support) to help high-risk patients connect to referrals. Organizations in the alignment track focused on aligning with community partners at the community level to build capacity for integrated health social care. Each track was linked to payment methods including start-up funds and additional funds for ongoing services (e.g., $2 per person per year for the awareness track, $86 dollars per person per year for the assistance track, and annual lump-sum payments of $350,000 per year for the alignment track).

#### Evaluation

CMMI will complete a federal evaluation of the Accountable Health Communities program by 2022 across the 29 funded sites. To facilitate evaluation activities, sites are required to follow rigorous research designs and standardized metrics. Sites in assistance tracks must randomize participants into an intervention group that receives both a list of community resources and intensive community service navigation or a control group that only receives a list of community resources. Sites in the alignment track must use a matched controlled design [19]. CMMI also set evaluation metrics, including total cost of care and health care utilization, including emergency department visits, inpatient admissions, readmissions, and utilization of outpatient services.

Early experiences have revealed a number of important insights related to process measures, infrastructure and implementation. In the first 750,000 completed screenings across programs across the country, 33% of patients reported at least one social need, with food being the most common need identified [20]. Eighteen percent of patients were eligible for navigation services, and 76% of eligible patients accepted navigation services. From the patient perspective, quantitative and qualitative studies have shown that patients find screening for social needs using the Accountable Health Communities tool appropriate and acceptable [21,22]. Case studies of providers identified that some perceive risks of collaboration between health and social services, including power dynamics between sector stakeholders, financial sustainability, and competition for employees [23].

### Medicaid 1115 Waivers Program

#### Description

While the federal government sets the core requirements and benefits covered by Medicaid, individual states can request the federal government waive specific Medicaid requirements and “approve experimental, pilot, or demonstration (state-level) projects…likely to assist in promoting the objectives of the Medicaid program” [24]. Since the 1960’s, these “Section 1115” waivers have been instrumental in giving state Medicaid agencies the flexibility to test innovative approaches aimed at improving care delivery and access to services to meet their beneficiaries’ unique needs.

Over the past decade, the Accountable Care Act and push towards value-based care models has catalyzed use of 1115 waivers for integrated care [25]. Sixteen states were granted 1115 waivers to pilot programs specifically focused on addressing social drivers of health (***Figure 1***), [26]. For example, California’s Whole Person Care (WPC) program is a $3 billion effort aimed to integrate physical health, behavioral health, and social service delivery for Medicaid beneficiaries who utilize acute and high-cost services [27]. WPC was granted 1115 waiver authority in 2016 because it used Medicaid funding to address social needs, which is beyond the scope of what is typically covered by Medicaid. During this five-year program, 25 WPC pilots across the state of California were approved to leverage data sharing infrastructure and strengthen cross-sector coordination, many partnering with county housing agencies, to improve health outcomes for their target population. Similarly, in 2018, North Carolina received an 1115 waiver to launch its Health Opportunities Pilots, a program that will use $650 million Medicaid health dollars to pay for non-medical services to address food insecurity, housing instability, transportation issues, and interpersonal violence across four regions in North Carolina [28]. These pilots will have standardized screening for social needs and pay for social services through value-based arrangements. The Healthy Opportunities Pilot will be supported by North Carolina’s NCCARE360, a technology solution for health and social service providers to refer and initiate appropriate services for individuals with identified needs across the state, and monitor the outcomes of the referral in order to optimize health outcomes [29]. Medicaid 1115 waivers have been instrumental in supporting pilot demonstration programs like these focused on delivery system reform to address social drivers of health across different states. ***Table 2*** lists other exemplar 1115 waiver demonstration programs that have included initiatives to address social determinants of health.

**Table 2:** Examples of SDOH-Related Activities Implemented Via 1115 Waivers.


STATE	EXAMPLE SDOH-RELATED ACTIVITIES

New York	Conducting household assessments and interventions for environmental health hazards related to asthma, indoor air quality, structural issues, fire safety, etc. [30]

Michigan	In response to the 2016 Flint Water Crisis, which exposed thousands of Flint, Michigan residents to lead-contaminated water. Provided targeted case management to children and pregnant women, funded lead abatement activities typically not covered by Medicaid, and connected families to community-based resources focused on early education programs, financial assistance, housing, etc. [31]

Rhode Island	Created “Accountable Entities” (AE), consisting of service providers and community-based organizations across health and social sectors, who are responsible for care of a defined population. AE’s conduct social needs screenings and are engaged in value-based payment arrangements to incentivize addressing social determinants. [32]

Oregon	Allows state Medicaid funding to be used on “health-related services” that improve quality such as case management, housing supports, and transportation services, as well as “community benefit initiatives” that focus on population-level interventions [33]

Illinois	Consolidated nine previously approved 1115 waivers to drive comprehensive change in service delivery. Includes measures such as connecting individuals to social supports like housing and building a robust workforce (e.g., leveraging community health workers to address cross-sector needs) [34]


#### Evaluation

The demonstration projects supported by 1115 waivers are evaluated by states and independent evaluators [35]. However, several issues have impeded rigorous state-led evaluations including a limited budget for evaluation, methodological challenges such as identifying appropriate comparison groups and inconsistent reporting of outcomes [36]. This has made it difficult for states to learn from each other’s approaches in a timely manner and for the federal government to identify innovations that have the potential to improve quality and costs.

Due to these concerns, the Centers for Medicare and Medicaid Services (CMS) has begun to conduct more robust evaluations on the national level and institute more rigorous evaluation criteria for states in recent years. First, in 2014, CMS began conducting meta-analyses to track performance and evaluate the outcomes of 1115 waivers implemented in similar domains across states [37]. From an integrated care perspective, key findings from these federal reports indicated that 1115 waiver programs have led to increased provider collaboration to support physical and behavioral health integration [38]. Furthermore, in 2019, CMS began providing states with evaluation tools tailored to their interventions and requiring states to report on performance measures that are specific to the type of demonstration project being evaluated (e.g., reporting on mental health services utilization for pilot projects on Substance Use Disorders) [35]. Thus, while evaluation remains a challenge, recent trends seem to indicate an increased focus on rigorous, transparent, and timely processes at the federal level.

### Medicaid Managed Care

#### Description

In 40 US states, the Medicaid program is administered by risk-based managed care organizations (MCOs), or private insurance companies that receive per-member, per-month payments from state Medicaid agencies to provide comprehensive services to their enrollees [39]. If MCOs spend more on services than they are paid by the state, they are at financial “risk” and undergo financial losses. From 2003 to 2018, as states have increasingly transitioned their Medicaid programs to the managed care model, the number of Medicaid beneficiaries enrolled in these risk-based managed care organizations has increased from 16 million to nearly 60 million [40]. This rise coupled with the growing evidence base linking unmet social needs with health has led Medicaid agencies to push MCOs to identify and address beneficiaries’ social needs as part of broader prevention efforts around reducing costs and improving health outcomes [41].

Between 2010 and 2020, 35 state Medicaid agencies have included efforts to integrate health and social care into managed care through contract requirements or incentives with the MCOs [39]. Six state examples are highlighted in ***Table 3***. While there is tremendous variation in states’ approaches, there are common elements and patterns. In contract requirements, states often embed addressing social needs within broader care coordination and care management requirements. States may require MCOs to screen individuals for social risks and develop care plans to identify needs related to food, housing, or employment [42]. Similarly, some may require using SDOH data to inform risk stratification frameworks for targeting interventions and allocating resources [43]. Others may require MCOs to coordinate with community-based organizations or government social services [44]. This coordination may be through designated “community service coordinators” who can ensure linkage to social services, a community advisory committee to develop strategies for improved integrated care, or contracts between MCOs and social services [43]. States may also create new value-based payment models, in which MCOs are given incentive payments or bonuses for meeting SDOH-related target measures [45].

**Table 3:** Examples of SDOH-Related Activities Supported Via Managed Care Contracts.


STATE	EXAMPLE SDOH-RELATED ACTIVITIES

New Mexico	Contract requires MCO to hire full-time housing supportive specialist to help beneficiaries access housing resources [46]

Arizona	Contract requires MCOs to invest six percent of profits into the community (e.g., community-based social services) [46]

Washington D.C	Contract requires MCOs to create quality improvement plan to address social determinants of health through targeted interventions [43]

Virginia	Contract requires MCOs to screen beneficiaries for social, economic, and housing needs, with referral mechanisms for community resources [46]

Michigan	Contract ties “pay for performance” bonuses for planning and reporting interventions to address SDOH (e.g., housing) [47]

Iowa	Contracts requires MCO to coordinate with state agencies (e.g., Juvenile Justice Services, Department of Education), and community-based organizations [48]


#### Evaluation

Formal process or outcome evaluations of Medicaid Managed Care contracts that attempt to integrate health and social care have not yet been conducted to our knowledge. Early survey and qualitative research has shown that state Medicaid agencies worry about including SDOH performance measures into contracts without well-validated measures or processes for assuring data quality. Similarly, Medicaid agencies have described how privacy and confidentiality policies limit sharing individual data across health and social service agencies (e.g., insurers, health systems, schools, justice system, non-profits, housing assistance, employment services), and thus limit cross-sector integration [49]. Medicaid managed care organizations reported that a lack of designated funding streams have made it difficult to adopt social interventions [41,50]. Researchers have an opportunity to partner with state Medicaid agencies and managed care organizations to co-develop studies to evaluate the implementation and effectiveness of efforts to address adverse SDOH within MCOs and identify best practices.

## Discussion

Policy reform and progress made over the last 10 years in the US have advanced the integration of health and social care. In this paper we focused on examples that target the 1 in 5 most socially vulnerable Americans insured through the Medicaid program, including one nationally funded demonstration and two state policy tools. These developments resulted from a policy opportunity that emerged from (1) new value-based payment models in healthcare that prioritize individual and population health outcomes over volume of services and (2) a growing evidence base linking social risk factors with health outcomes. Implementation of these new policy initiatives have required a complex web of actors including government health agencies, private payers, health care organizations, and community-based organizations. While research on integrating health and social care in the US has focused on small interventions implemented in individual clinics and hospitals and is limited by poor study quality and a focus on process measures [51,52], efforts from these broader policy initiatives have reached millions of Americans. Comprehensive evaluation, with a focus on implementation and effectiveness, is still needed.

Although the US has historically lagged in efforts to integrate health and social care compared to other high-income countries, the US is not alone in its recent commitment to this area. To identify cross-cutting learnings across various US initiatives and contextualize them globally, we applied an adapted SELFIE framework for integrated care for multi-morbidity [53]. Developed from the World Health Organization’s building blocks for health systems, this framework aims to aid the development, implementation, description, and evaluation of integrated care programs. We use the framework here to guide a more conceptual discussion of policy and program components for integrated health and social care. Specifically, we draw on international literature to discuss five main framework concepts: (1) governance and culture, (2) financing, (3) service delivery, (4) workforce, and (5) information & research.

### Governance & Culture

Over the last decade, federal and state governments in the US have demonstrated a newfound and robust commitment to health and social care integration [54]. However, given that service delivery is largely driven by the private hospitals and clinics in the US, policy action from public actors has primarily focused on changes in financing and payment structures. In addition, given the Medicaid program is administered at the state level, the federal government has focused on giving states flexibility in the types of service delivery models each state employs and what benefits are afforded to beneficiaries. This has resulted in a “bottom up” or decentralized approach to health and social care integration, in which the government provides incentives and flexibility for care re-design, and private actors and community-based organizations implement changes in services, workforce, and health information technology [55].

This is in contrast to the United Kingdom (UK) and Germany, which have taken a more “top down” approach, led by the central government, by aligning goals and setting standards across medical and social service agencies [55]. For example, in 2013, the UK government partnered with health and social care organizations to create a national policy framework called “Integrated Care and Support: Our Shared Commitment” that outlined a shared vision for reform and available government resources [56]. While the US’s predominantly “bottom up” healthcare governance model allows for integrated care efforts to be better tailored to local needs, lack of alignment on overarching goals at a national level may hinder collective action on building the workforce or information technology to implement successful strategies more broadly.

Often a hybrid approach which combines the “top down” and “bottom up” governance structures is the most effective. This model ensures that there is national guidance to drive integrated care but it is not overly prescriptive as to preclude adapting models to local context [57,58]. Across approaches and countries, efforts to improve integrated care should actively engage patients, local communities, and the social care sector in the co-design of new care delivery models [59]. If the health sector alone leads integrated care reforms, it could unintentionally “medicalize” SDOH, in which social needs are viewed like a pathology to diagnose and treat at the point of care, with little acknowledgement of the root causes.

### Financing

Policy efforts to finance integrated health and social care in the US have focused on providing flexibility to use healthcare dollars to address SDOH and social needs through capacity-building and direct service delivery. This approach resulted from the US’ lopsided health-to-social service spending ratio. That is, for every $1 spent on health care in the US, $0.90 is spent on social services [14]. In contrast, in OECD countries, for every $1 spend on healthcare, an average of $2 is spent on social services. As a result, federal healthcare dollars in the US are funding cross-sector partnerships between health and social services, as seen in the “Accountable Health Communities” model. At the state level, Medicaid agencies are using payment levers to incentivize or reward private healthcare payers to invest in social care interventions. The state of North Carolina is even using Medicaid healthcare dollars to directly pay social service providers (e.g., food banks, housing agencies, etc.) [28]. Some experts worry that health care-centered integration efforts may inadvertently medicalize social care [60] or overburden underfunded social services [10]. Additionally, financing interventions that target individual-level social needs fail to address the upstream, community-level or systemic root causes that lead to health-related inequities [61].

In contrast, other countries have experimented with more bi-directional financing mechanisms between health and social services. Models may include aligning budgets across sectors to meet agreed-upon objectives; pooling funds and management staff across sectors into common buckets; or even integrating health and social services under a single management body [62]. In Sweden, the Norrtalje model brought together the local health care governance structure (“Stockholm County Council”) and local social care governance structure (“Norrtalje Local Authority”) to form a new join Governing Committee that pooled funding from both original structures [63]. The US should consider this model and other international examples, as it moves to adopt more advanced budgeting tools for cross-sector integration [64].

### Service Delivery

In the US, efforts to integrate health and social care have mainly focused on identifying and responding to individual patients’ social needs. All three Medicaid policy examples described in this paper include requirements or incentives for standardized screenings for social needs. Once patients are screened, health care organizations can help patients connect to social services [51]. These individual-level programs have been complemented by broader collaborations between healthcare organizations and social services, such as medical-legal partnerships and clinic-based food pantries [12].

Globally, this model of linking patients with non-medical services for social needs has been coined “social prescribing” and is often associated with primary care [65]. In March 2019, 11 countries celebrated the world’s first Social Prescribing Day, including Finland, UK, Brazil, and Canada [66]. There are important distinctions between American social prescribing schemes and those from other countries. The US has developed standardized screening questions that have been used across primary, secondary, and tertiary care settings [67]. Simple and standardized approaches allow for patient self-administered screening; scalable pathways for responding to reported needs; population-level tracking of social needs; and consistent measures for program evaluation and research. Common questions in the US include screening for transportation, food insecurity, housing instability, utilities, and interpersonal violence [68].

Other international models often implement broader individually-tailored approaches. Patients may be asked about overall social and mental well-being, and social prescribing staff may co-create strength-based care plans with patients [69]. Thus, while US social prescribing initiatives supported through Medicaid mostly target basic material needs (e.g., food and housing), international models more often include more holistic referrals (e.g., exercise, volunteering, parks, recreation centers, and art museums) [70]. Despite policy support globally, social prescribing initiatives within and outside individual countries greatly vary in intervention components, intensity, target populations, and settings. More international research is needed to study the effectiveness and value for money of social prescribing programs, particularly under different financing and governance structures.

### Workforce

The US policy efforts have not outlined a comprehensive workforce strategy for integrating health and social care, including for the Medicaid population. The workforce used for social prescribing activities in the US has been heterogenous and include both the traditional health care workforce (e.g., doctors and nurses) and the social care workforce (e.g., case managers, social workers, and volunteers) [71]. Although research of workforce models suggest interprofessional care teams in clinical settings and community health workers in community and home-based settings could be possible in the US [14], barriers to reimbursement remain a challenge.

In contrast, other countries have focused on policies to deploy a standardized workforce to integrate health and social care. For example, the UK National Health Service (NHS) aims to fund 1,000 social prescribing “link workers” to work in primary care practices by 2021 [72]. The NHS is working to ensure that link workers have clinical supervisors, access to peer support and regional learning coordinators, and online training packages [72]. Similarly, Germany has instituted a training program at the national level for nurses to gain the skills and expertise to serve as case managers and care coordinators to support integrated care [55]. In contrast, in the US such roles are hired and trained at the local level. Future efforts to develop a workforce to integrate health and social care in the US should look to such global examples to gain insight into recruitment, training, and team integration.

### Information & Research

Emerging evidence in the US has focused on local health-system interventions to integrate health and social care rather than the policies that support them [73]. Studies in the US have demonstrated the feasibility and patient acceptability of screening for social needs [21,22,74,75], as well as the need for further studies on the psychometric and pragmatic properties of various screening tools [76]. More research in the US and internationally is needed to evaluate the effectiveness and cost-effectiveness of social prescribing programs that link patients with community resources, particularly under different financing and governance structures [52,68]. However, evaluating integrated care efforts like social prescribing presents many challenges. First, determining the impact of these programs can be difficult given lack of alignment on measures and measure specifications [77,78,79]. Historically, there has been little standardization of social needs screening questions or the measures for evaluating the “success” of interventions (e.g., process measures, social care impacts such as income or food insecurity, health and well-being impacts, healthcare costs and utilization impacts, provider outcomes related to burnout or efficiency). Certain initiatives in the US, such as the Gravity Project initiated by the University of California-San Francisco, are making headway in standardizing how social risk data is captured in electronic health records (EHR) [80]. Second, few studies have used rigorous study designs or evaluate impact beyond process measures or improvements in specific social needs or subpopulations (e.g., high healthcare utilizers). At the policy level, governments globally can support national evaluation frameworks and better fund research to fill evidence gaps.

Another necessary component of analyzing patient and population-level data is appropriate information technology. In 2009, the US passed a new law to increase the adoption of EHRs among healthcare providers and systems [81]. Widespread uptake of EHRs created an opportunity to routinely collect measures of patients’ social risks in the clinic setting. However, there has been little policy guidance on which SDOH screening tools to embed in the EHR or which codes to use to classify social risks [82]. Furthermore, while health systems can document and aggregate data related to social risk screening and referrals to community resources, social service providers often do not have access to patients’ EHRs due to privacy and confidentiality laws. These challenges fragment care, and prevent the ability to generate real world evidence or evaluate innovative models without external funds for research or additional investment on measurement.

To overcome the limitations in US informational continuity across settings, recent efforts have created new technology platforms that both health and social service partners can access to make bi-directional referrals and monitor care management [83]. Medicaid state agencies or Medicaid managed care organizations can license these software products from private companies or foundations. For example, as part of its Medicaid reform, North Carolina has launched NCCARE360, “a statewide coordinated care network” which contains a resource directory and referral system that is accessible to health and social service providers, and an ability to track outcomes [29]. Implementation research is in its earliest stage and state-level evaluation is likely to align with the previously mentioned Healthy Opportunities Pilots.

Similar pushes for digital platforms to facilitate cross-sector integration have emerged in the UK [84]. Private companies have started to contract with health care organizations and community-based organizations to implement their platforms across a range of providers [85,86,87]. Additionally, some health information technology efforts in the UK have focused on increasing interoperability between healthcare systems, enabling national audits and secondary analysis of public health systems. In 2017, the NHS chose eight regions across England to transition to accountable care systems (ACS) to better integrate and coordinate care at the community level [88]. As part of these efforts, each ACS has designed its own digital shared care record that is interoperable across different NHS entities and accessible to a patients primary, community, secondary and social care providers. While information technology efforts in the US mirror many of the efforts in the UK to integrate care, future efforts in the US should focus on increasing data interoperability across sectors and health systems.

### The Decade Ahead

***Table 4*** summarizes the main lessons learned from the three integrated health and social care policy initiatives over the last decade and highlights priority areas for future work. The start of the 2020s, with the COVID-19 pandemic and a new presidential administration, has created a new opportunity to build upon and sustain recent progress towards integrated care [89]. The COVID-19 pandemic has further exposed the role that social inequalities play in shaping health inequalities [90]. Americans from low-income communities have been more likely to contract and become hospitalized from COVID-19, and suffer from the pandemic’s economic repercussions, such as unemployment and increased food insecurity [89,91]. As the country realizes the need for a coordinated response to intertwined social and health challenges, the new Democratic administration can prioritize integrated health and social care in their broader “Build Back Better” strategy, already demonstrated by their interest in adding 150,000 community health workers to underserved communities and creating a new Public Health Job Corps to address SDOH [92]. While previous policy efforts for integrated care have largely been state-driven, the new administration could provide leadership to create a bold national strategy around data infrastructure, workforce, financing, and service delivery [93].

**Table 4:** Lessons Learned.


INTEGRATED CARE DOMAIN	LESSONS LEARNED AND POLICY IMPERATIVES IN THE US

Governance & Culture	Integrating health and social care requires buy-in from front-line clinicians, insurers, and government actors. Future initiatives should explore how to better engage social service providers and local communities in integrated care reforms.

Financing	New flexibility granted from policymakers has enabled health care organizations to use healthcare dollars to fund social care interventions. Future efforts should explore bi-directional funding mechanisms that align priorities across health and social sectors, and pool funds to achieve shared outcomes.

Service Delivery	The health sector has tested and evaluated new interventions to screen patients for social risks and refer them to social services. Ongoing implementation efforts should consider how services can be tailored to local contexts and clinical settings in order to maximize patient and provider adoption. Additional research on comparative and cost effectiveness can help identify which social risks and patient populations should be prioritized in scale-up efforts.

Workforce	Healthcare organizations have successfully deployed a variety of individual workers and interprofessional teams to address patients’ social risks. However, the US may benefit from a national strategy to develop and fund a new workforce committed to integrating health and social care, as seen in Europe.

Information and Research	Increased uptake of electronic health records has enabled capture of SDOH data, and new technology platforms have allowed for referrals from healthcare to social services. Future initiatives should address the lack of cross-sector data sharing due to confidentiality laws and poor interoperability.


## Conclusion

Over the last decade, new policies in the US have catalyzed initiatives to test innovative integrated health and social care models, with the ultimate goal of improving population health and decreasing costs. The examples from the US Medicaid insurance program highlighted in this paper describe the country’s progress in an international context. Continued investment is needed into each building block to ensure a sustainable approach to integrated care. Cross-nation collaboration is needed to globally advance policies and research that improve population health and address individuals’ needs.
